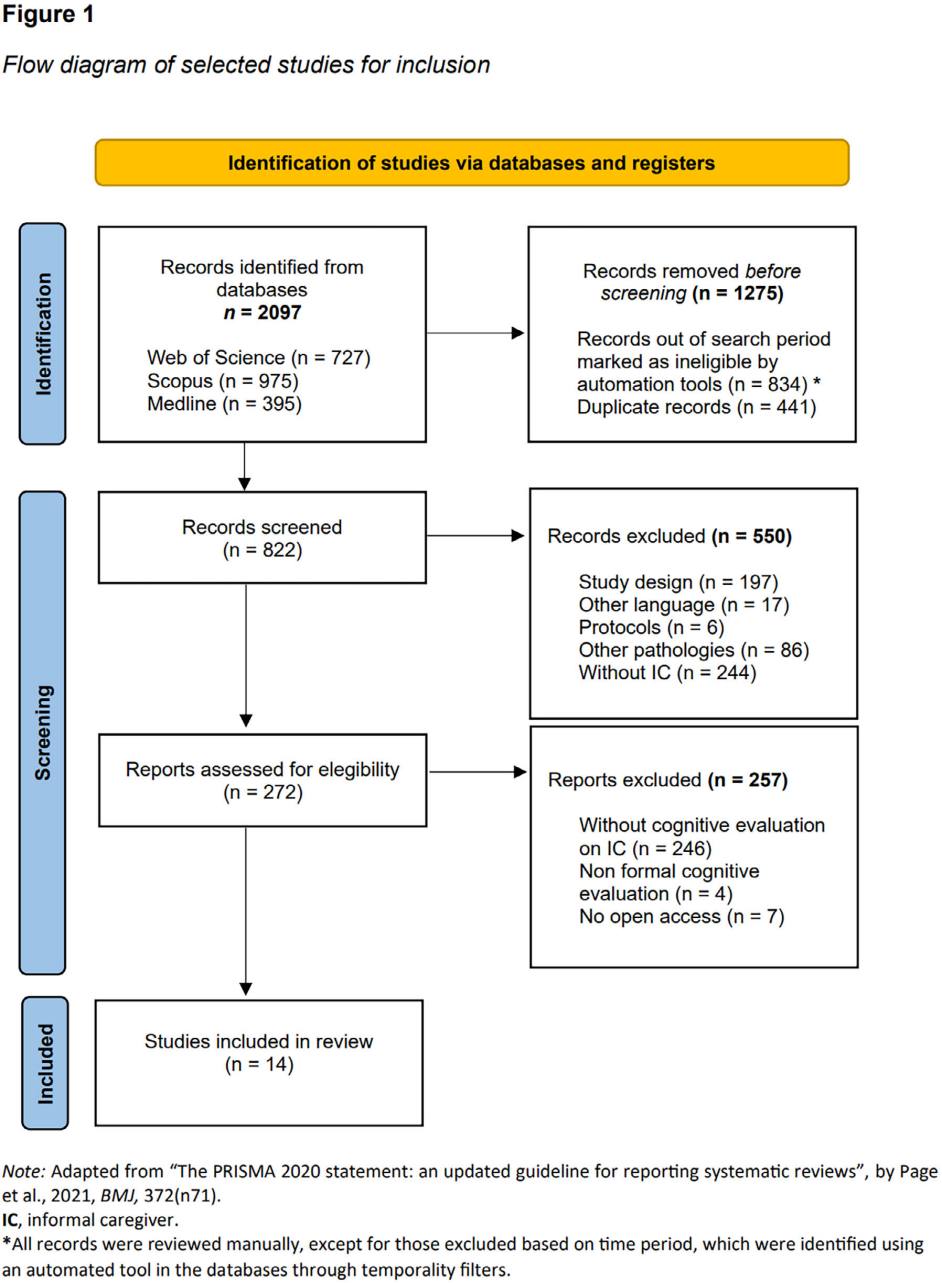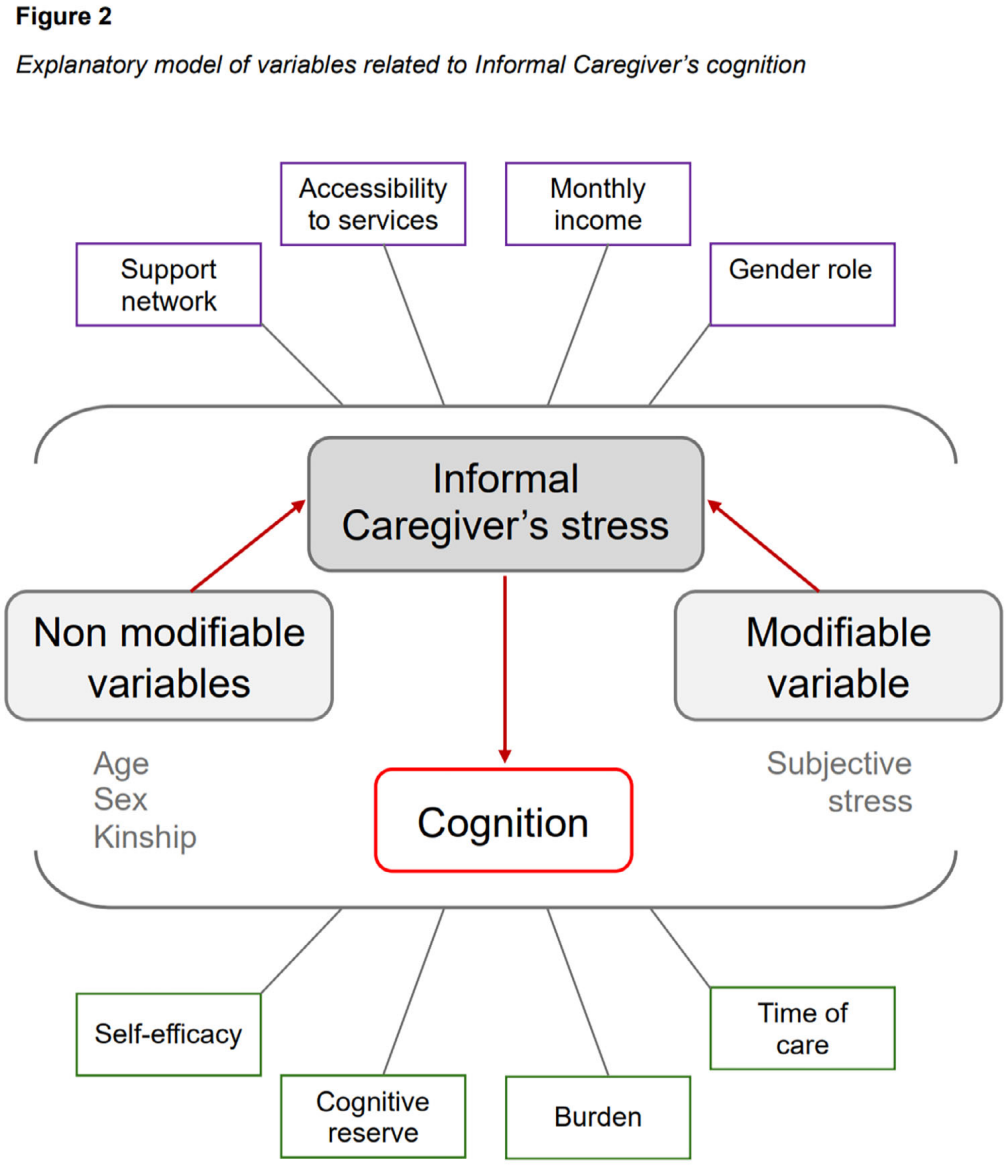# Is caregiving for someone with neurocognitive disorder a risk factor for developing cognitive decline? A systematic review of cognitive function among informal caregivers

**DOI:** 10.1002/alz70858_106172

**Published:** 2025-12-26

**Authors:** Valeria Macias‐Aguinaga, Joaquín Mateu‐Mollá

**Affiliations:** ^1^ AXXIS Hospital de Especialidades, Quito, Pichincha, Ecuador; ^2^ Valencian International University, Valencia, Comunidad Valenciana, Spain

## Abstract

**Background:**

Informal caregivers represent an important role within caregiving due to the increase in the number of elderly people living with neurocognitive disorders requiring assistance. The demands associated with this role have an impact on the caregiver's well‐being and quality of life; however, knowledge on the effects on cognition is scarce. For this reason, we propose to gather scientific evidence on cognitive performance of informal caregivers, as well as demographic and clinical variables associated with the sample, to assess whether this role represents a risk factor for cognitive decline.

**Method:**

A systematic review of information was carried out using the PRISMA protocol through the following databases: Web of Science, Scopus and PubMed from 2014 to December 1st, 2023.

**Result:**

A total of 14 empirical articles were analyzed and it was shown that 78.57% of studies reported cognitive decline, 14.29% better cognitive status and 7.14% showed no significant differences between informal caregivers and non‐caregivers. The majority of caregivers were older, female and spouses. Chronic stress is related to informal caregiver's cognitive performance, especially in executive function.

**Conclusion:**

Being an informal caregiver of a person living with neurocognitive disorders does not represent an isolated risk factor in caregiver's cognition; however, the interaction of the different variables associated with caregiving influences the outcome and increases the likelihood of cognitive decline, especially if these are not addressed in a timely manner.